# An efficient ELISA protocol for measurement of SARS-CoV-2 spike-specific IgG in human plasma and serum samples

**DOI:** 10.1016/j.mex.2024.102596

**Published:** 2024-02-05

**Authors:** Gwenllian A. Appeltrath, Janine Parreuter, Monika Lindemann, Hannes Klump, Christina B. Karsten

**Affiliations:** a Institute for the Research on HIV and AIDS-associated Diseases, University Hospital Essen, University of Duisburg-Essen, Essen, Germany; b Institute for Transfusion Medicine, University Hospital Essen, University of Duisburg-Essen, Essen, Germany; c Institute for Transfusion Medicine and Cell Therapeutics, University Hospital RWTH Aachen, Aachen, Germany

**Keywords:** SARS-CoV-2 spike, ELISA, IgG, SARS-CoV-2 Spike-Specific IgG ELISA

## Abstract

Here, we describe a protocol for the detection of severe acute respiratory syndrome coronavirus 2 (SARS-CoV-2) spike-specific immunoglobulin G (IgG) by enzyme-linked immunosorbent assay (ELISA). The protocol was developed with a keen focus on optimizing several key parameters, including antigen coating concentration, antibody and sample dilutions, and assay development time. The final protocol features the following characteristics:

• The capability to detect SARS-CoV-2 spike-specific IgG in both plasma and serum samples.
• A streamlined procedure that requires only 1 hour and 20 minutes of hands-on time.
• Reliable assay performance, with a remarkable sensitivity of 98.1 % and specificity of 99.5 %.

## Graphical abstract

**Figure:**
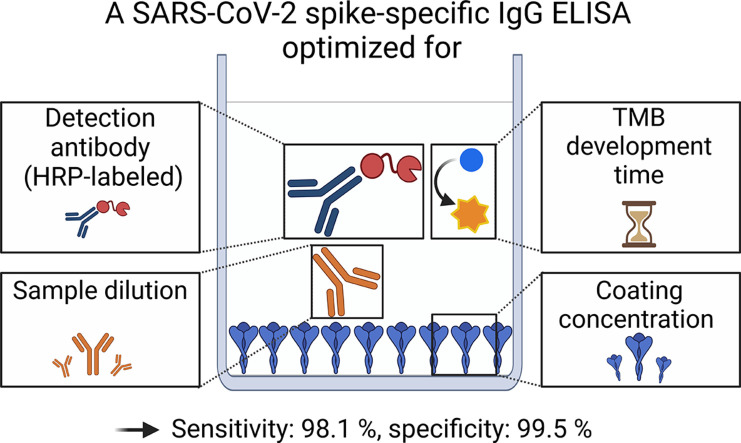

Specifications table

**Table:** 

**Subject area**	Immunology and Microbiology
**More specific subject area**	Diagnostics
**Name of your method**	SARS-CoV-2 Spike-Specific IgG ELISA
**Name and reference of original method**	n.a.
**Resource availability**	**Reagents, equipment, consumables, software** **Reagents** *Commercially available* • Sodium hydrogen carbonate, Sigma-Aldrich, CAT# 401676 • Phosphate-buffered saline (PBS), Carl Roth, CAT# 9150.1 • Sulfuric acid, Sigma-Aldrich, CAT# SIG-258105-1L-PC • Tween 20, Sigma-Aldrich, CAT# P9416 • 1-Step Ultra-TMB substrate, Thermo Scientific, CAT# 34028 • Bovine serum albumin (BSA), Roche Diagnostics, CAT# 10735086001 • Soluble stabilized SARS-CoV-2 Wuhan-Hu-1 spike protein produced by plasmid transfection into FreeStyle 293-F cells (plasmid: CAT# NR-52394, BEI Resources; ready-made protein: CAT# NR-52397, BEI Resources) • Sheep serum, Sigma-Aldrich, CAT# S22-100ML • Goat anti-human IgG (Fc) cross-absorbed-HRP (detection Ab), Sigma-Aldrich, CAT# SAB3701283-1mg, RRID:AB_2936313
	*Self-prepared buffers and solutions* Stock solutions of PBSA and PBSA-S should be kept sterile and stored at 4 °C. • 0.1 M sodium hydrogen carbonate (pH 8.6) • 1 N sulfuric acid • PBS with 0.05 % Tween 20 (PBS-T5) • PBS with 5 % BSA (PBSA) • PBSA with 0.01 % Tween 20 (PBSA-T1) • PBSA with 20 % sheep serum (PBSA-S) *Assay controls* • Positive control: SARS-CoV-2 IgG^high^ serum or plasma sample. If not available, a positive control can be generated by dilution of SARS-CoV-2 spike specific Ab, e.g. CR3022 (expression plasmid set used for production in FreeStyle 293-F cells: CAT# NR-53260, BEI Resources; ready-made Ab: CAT# NR-52481, BEI Resources, RRID:AB_2936472), in non-reactive serum or plasma. • Negative control: serum or plasma drawn from healthy donors before 2019. **Equipment** *Utilizing a plate washer minimizes variation in assay results and is the preferred method over manual washing of ELISA plates using a multichannel pipette.* • Class II biological safety cabinet, Thermo Scientific, CAT# 51025411 • Eppendorf Research plus pipettes: ○ 30 – 300 µL, 12-channel, CAT# 3125000060 ○ 2 – 20 µL, 1-channel, CAT# 3123000039 ○ 20 – 200 µL, 1-channel, CAT# 3123000055 ○ 100 – 1,000 µL, 1-channel, CAT# 3123000063 • Plate washer HydroSpeed, Tecan, CAT# 30054550, RRID:SCR_023457 • Plate reader Spark, Tecan, CAT# 30086376, RRID:SCR_021897 **Consumables** • Pipette tips with filter ○ 10-20 µl, TipOne, CAT# S1120-3710 ○ 20-200 µL, Biosphere, CAT# 70.1189.215 ○ 100-1000 µl, TipOne, CAT# S1122-1730 • Single use reservoir, Integra, CAT# 4312 • Plates: ○ 96-well plates, polystyrene, transparent, U-bottom, Greiner Bio-One, CAT# 650101 ○ 96-well ELISA plates, polystyrene, transparent, F-bottom, MaxiSorp, Nunc, Thermo Scientific, CAT# 442404 • 50 mL tubes, Greiner Bio-One, CAT# 227261 • EASYseal plate sealer, transparent, Grainer Bio-One, CAT# 676001 **Software** • SparkControl (version 3.1), Tecan, included with Plate washer • GraphPad Prism 9 (version 9.5.0), Dotmatics

**Method details**

## Introduction

In 2019, the novel coronavirus known as severe acute respiratory syndrome coronavirus 2 (SARS-CoV-2) emerged, causing the widespread coronavirus disease 2019 (COVID-19) pandemic [1]. At the beginning of the pandemic, laboratory resources, including tests to measure SARS-CoV-2 antibody (Ab) responses, became limited and were primarily allocated to facilities involved in the immediate management of the health crisis [2]. To enable us to carry out SARS-CoV-2 research specifically relevant for human immunodeficiency virus (HIV) diagnostic and the community of individuals living with an HIV infection [3], we developed an enzyme-linked immunosorbent assay (ELISA) protocol for the detection of SARS-CoV-2 spike specific immunoglobulin G (IgG) in serum or plasma samples. In a nutshell, the protocol involves coating 96-well plates with SARS-CoV-2 spike protein in sodium hydrogen carbonate on the first day, followed by overnight incubation at 4 °C. Subsequently, the plates are washed and blocked for one hour at room temperature. After diluting the samples, they are added to the plate and incubated for a duration of 2 hours. Afterwards, the plate is washed, and an horseradish peroxidase (HRP)-labeled detection Ab specific to the crystallizable fragment (Fc) region of human IgG is added, followed by another one-hour incubation period. The plates are washed again, and 3,3′,5,5′-tetramethylbenzidine (TMB)-substrate is added for development, which is stopped using sulfuric acid.

## Procedure

It is imperative for all experimenters to adhere to all applying regulations concerning necessary precautions, personal protective equipment, and laboratory sample handling. The ELISA should be conducted at room temperature, unless stated otherwise, and without any shaking. Throughout the incubation periods, it is essential to seal the plate with a plate sealer. To optimize efficiency, use a multichannel pipette and a reservoir whenever feasible. Similarly, for time-saving purposes, prepare sample dilutions in cell culture plates rather than 1.5 ml reaction vessels.

## Day 1

### Antigen immobilization

1. Prepare a 5 µg/mL final concentration of SARS-CoV-2 spike protein by diluting it in a 0.1 M sodium hydrogen carbonate solution (pH 8.6).
2. Add 100 µl of the diluted protein per well of a 96-well ELISA plate.
3. Incubate the plate overnight at 4 °C.

## Day 2

### Blocking of ELISA plates

If an automated plate washer is not available, you can use a multichannel pipette for plate washing. In that case, remove the washing buffer by flicking the plate after each wash and gently dry the plate on a paper towel before proceeding.

4. Wash the ELISA plate 4 times with 400 µl of PBS-T5 using an automated plate washer and blot it dry.
5. Add 360 µl PBSA-T1 per well.
6. Incubate the plate for 1 h at room temperature.

### Addition of samples

When handling human clinical samples (steps 7, 9, and 11), use a biosafety cabinet. For a project involving serum and plasma samples from SARS-CoV-2 convalescent individuals, we found that a dilution of 1:200 provides the best sample resolution.

7. During step 6, vortex the samples and dilute in PBSA-S in a 96-well cell culture plate.
8. Wash the ELISA plate 4 times with PBS-T5 and blot it dry.
9. Add 100 µl of sample dilution to each well of the ELISA plate.
10. Incubate the ELISA plate for 2 h at room temperature.

### Addition of detection Ab

11. Wash the ELISA plate 6 times with PBS-T5 and blot it dry.
12. Briefly mix the detection Ab by vortexing and dilute 1:20,000 in PBSA.
13. Add 100 µL of the diluted detection Ab to each well.
14. Incubate for 1 h at room temperature.

### Development and measurement

15. Wash the ELISA plate 6 times with PBS-T5 and blot it dry.
16. Develop by adding 100 µL of 1-Step Ultra-TMB substrate.
17. Incubate the plate in the dark for 30 min at room temperature.
18. Stop the reaction by adding 100 µl of 1 N sulfuric acid to each well.
19. Read the absorbance of each well at 450 nm with reference wavelength 570 nm using a plate reader.

### Time taken

The execution of the ELISA according to the protocol typically takes approximately 5 hours and 30 minutes (excluding overnight incubation), with a hands-on time of around 1 hour and 20 minutes. These estimates assume that preparations for subsequent steps are carried out concurrently with ongoing incubations.

**Table:** 

Procedure	Step	Hands-on time	Incubation time
**Immobilization of antigen**	Coating (1–3)	10 min	Overnight
**Blocking of ELISA plates**	Wash (4)	2 min	-
	Blocking (5-6)	5 min	1 h
**Addition of samples**	Preparation of sample dilutions (7)	15-25 min	-
	Wash (8)	2 min	-
	Sample application (9-10)	5 min	2 h
**Addition of detection Ab**	Wash (11)	10 min (manual wash)	-
	Dispensing of detection Ab (12-14)	5 min	1 h
**Development and measurement**	Wash (15)	3 min	-
	Addition of TMB substrate (16-17)	5 min	30 min
	Stop of development (18)	5 min	-
	Measurement (19)	5 min	-

## Anticipated results

The SparkControl software of the Tecan Spark plate reader will generate an excel sheet containing the measurement results. The plate reader accurately detects optical density (OD) values between 0 and 4. In cases where the signal exceeds the detection limit, the plate reader displays "over" instead of a specific value. The ELISA assay readings are specifically measured at 450 nm, and it is essential for all assay values to fall within the designated detection range. If any values surpass the upper limit, it is necessary to repeat the ELISA with higher dilutions of the samples to prevent signal saturation. To determine the background signal of the assay plate, the reference wavelength of 570 nm is measured. It is crucial for these reference values to remain below 0.01. After subtraction of the reference wavelength signal from the sample signals, assay results are analyzed by GraphPad Prism 9. To ensure the validity of the assay, certain criteria must be met: a strong positive control should yield an OD of ≥1.5, while the negative control should not exceed an OD of ≤0.3. A sample is considered positive for SARS-CoV-2 spike IgG if its value exceeds the negative control signal by at least two-fold.

## Troubleshooting

If the quality of the assay does not meet the expectations, please refer to the following suggestions regarding common sources of errors that may occur during the conduction of ELISAs.

**Table:** 

Problem	Possible reason	Solution
**Weak or no signal**	Reagent issues	Ensure reagents are warmed up to room temperature before use; check expiration dates and proper storage conditions; recalculate all prepared dilutions, especially for the detection Ab
	Samples too diluted	Perform sample titrations; check calculations for sample dilutions for errors
	Coating Ab not bound to assay plate	Confirm the use of an ELISA plate for coating, not a tissue culture plate
	Ab coating damaged	Avoid scratching of the well bottom during pipetting; check plate washer settings and adjust height of needles if necessary
	Measurement of incorrect wavelength	Adjust plate reader absorbance settings to the correct wavelength
**Too high signal**	Insufficient washing	Increase number of washes
	Incubations too long	Use a timer to adhere to incubation times
	TMB exposed to light	Store TMB protected from light at all times
**Inconsistent results**	Edge effects	Ensure plate sealers are fully attached to prevent evaporation
	Sample carry-over	Change pipette tips between samples; avoid touching the ELISA plate with the pipette; use a plate sealer
	Irregular washing	Clean the dispenser of the plate washer
**Poor data reproducibility**	Variation in incubation times between plates	Keep incubation times consistent across different assay plates
	Inconsistent sample dilutions	Use the same sample dilutions for all experiments

## Method validation

**Fig. 1:**
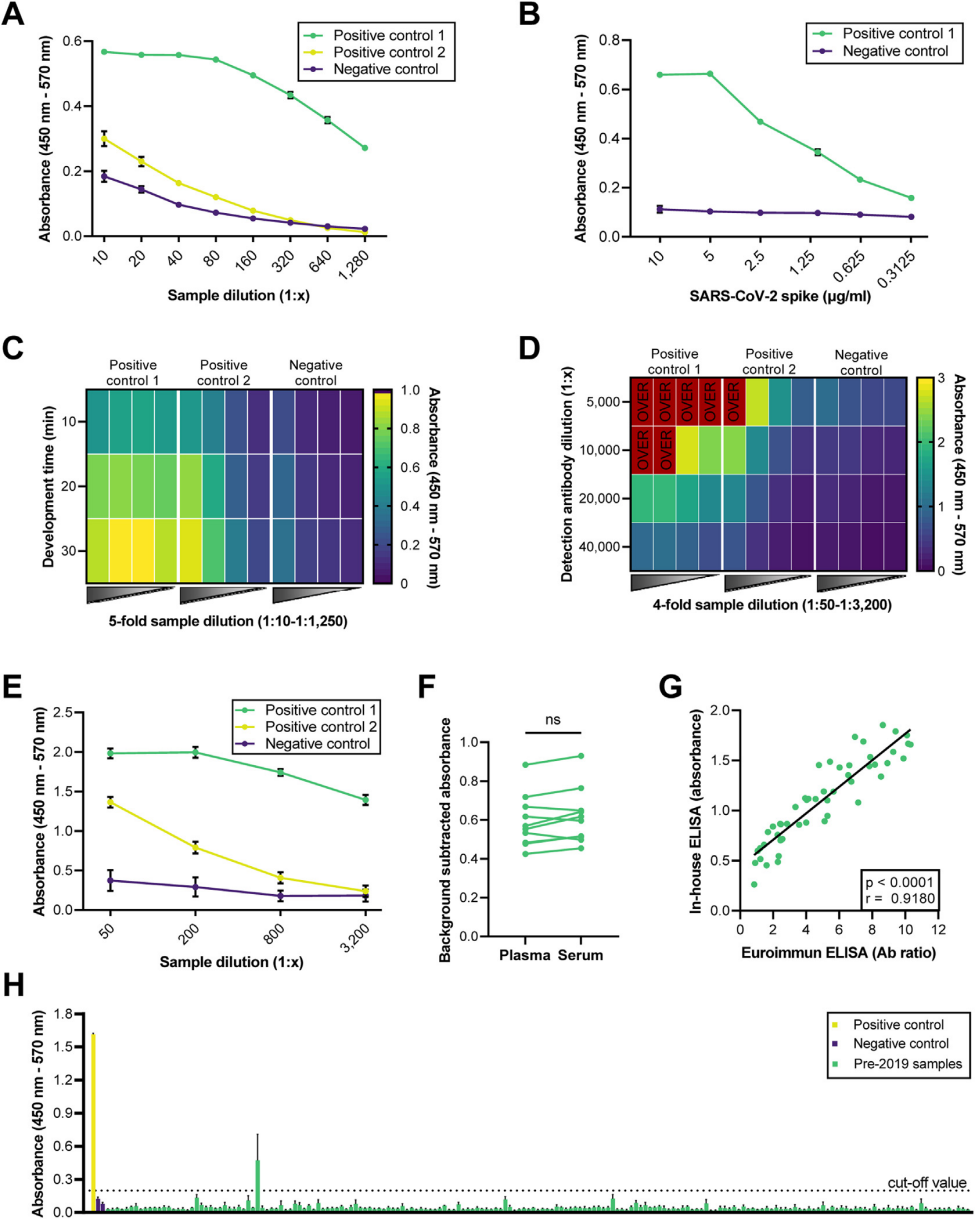
Characterization and optimization of an in-house ELISA protocol for the detection of SARS-CoV-2 spike-specific IgG (A) Results of the initial non-optimized ELISA protocol for detecting SARS-CoV-2 spike-specific IgG Abs in human plasma samples. (B) SARS-CoV-2 spike protein concentrations in the ELISA coating buffer were systematically varied. (C) Results of ELISA development times of 10, 20, and 30 minutes are shown. (D) Different dilutions of the detection Ab in the ELISA protocol were examined. Raw values exceeding the detection limit of the plate reader were labeled as "OVER". (E) Depiction of the sample resolution for a detection Ab dilution of 1:20,000. (F) Matching plasma and serum samples of 9 SARS-CoV-2 spike IgG+ individuals were analyzed by ELISA. Potential differences between groups were determined by a two-sided paired t-test (α = 0.05). ns = non-significant. (G) Human plasma samples of individuals (n = 52) with diagnostically confirmed SARS-CoV-2 spike-specific IgG Abs were tested using the optimized in-house ELISA protocol to determine assay sensitivity. The data from all reactive samples in the in-house ELISA (n = 51) were plotted against the results of the in-vitro diagnostics certified Euroimmun anti-SARS-CoV-2 ELISA (IgG). (H) To evaluate the specificity of the in-house ELISA, serum samples (n = 185) collected prior to the corona pandemic in 2018 were analyzed. The dashed line represents the cutoff of 2-fold over background, above which samples are considered reactive. All assays were performed in duplicates or triplicates with at least two independent repetitions. All graphs show the grand mean. Any error bars depict the standard error of the mean. Pictures were generated using GraphPad Prism 9.

In order to develop the ELISA protocol, we systematically evaluated and adjusted several key assay parameters, including the coating concentration of soluble SARS-CoV-2 spike, assay development time, and dilutions of the detection Ab and human plasma samples. Initially, the draft protocol employed a coating concentration of 10 μg/ml of SARS-CoV-2 spike, a 10-minute assay development time, and a detection Ab dilution of 1:40,000. While these conditions already allowed for the distinction of SARS-CoV-2 spike reactive and non-reactive human plasma samples, low sample dilutions of 1:80 or less were required to separate a sample with medium reactivity from the negative control (Fig. 1A).

To improve the ELISA protocol, essential assay parameters were subsequently optimized, and improved conditions were implemented as protocol modifications for subsequent refinements. First, we performed a titration experiment using 0.3-10 μg/ml of SARS-CoV-2 spike and a sample dilution of 1:100 (Fig. 1B). This experiment demonstrated that a coating concentration of 5 μg/ml of SARS-CoV-2 spike is sufficient to detect highly reactive samples without signal loss (Fig. 1B). Next, we tested development times of 10, 20, and 30 minutes and found that a development time of 30 minutes provided the optimal signal-to-noise ratio (Fig. 1C). Subsequently, we assessed the ideal dilution of the detection Ab and determined that dilutions of 1:5,000 and 1:10,000 generated signals above the detection limit for SARS-CoV-2 IgG+ plasma samples, while a dilution of 1:40,000 resulted in poor signal resolution (Fig. 1D). Consequently, we chose a detection Ab dilution of 1:20,000 for the final protocol. Finally, titrating the plasma samples using the optimized ELISA protocol revealed the highest sample resolution at a sample dilution of 1:200 (Fig. 1E).

To evaluate the optimized ELISA protocol for the measurement of SARS-CoV-2 spike specific IgG, we investigated its suitability for the analysis of human serum samples (Fig. 1F). For that purpose, matching plasma and serum samples of 9 individuals containing SARS-CoV-2 spike-specific IgG were tested in parallel using negative plasma and serum controls, respectively. The results showed comparable signals between plasma and serum samples after background subtraction, indicating that the ELISA protocol is applicable for analyzing both types of biospecimens. In order to assess the assay's sensitivity, we analyzed plasma samples (n = 51) from individuals with known SARS-CoV-2 spike-IgG titers, as determined by an in-vitro diagnostics certified commercial ELISA (Euroimmun-Anti-SARS-CoV-2-ELISA (IgG)), using our in-house ELISA (Fig. 1G). In the self-developed ELISA, 50 out of 51 plasma samples exhibited reactivity, resulting in an assay sensitivity of 98.1 %. Furthermore, the results of both ELISAs positively correlated (r = 0.9180, p < 0.0001). Assay specificity was determined to be 99.5 % using serum samples (n = 185) collected in 2018 prior to the SARS-CoV-2 pandemic (Fig. 1H). In summary, based on these findings, we conclude that the established protocol enables efficient and reliable detection of SARS-CoV-2 spike-specific IgG in both human plasma and serum samples.

## Ethics statements

This study was approved by the Institutional Ethics Committee of University of Duisburg-Essen (identifier: 20-9459-BO (date of approval: July 15, 2020); 20-9225-BO (date of approval: April 2, 2020). The study was conducted in accordance with the code of ethics of the World Medical Association (declaration of Helsinki) for experiments involving humans and the uniform requirements for manuscripts submitted to biomedical journals. Written informed consent was obtained from all subjects involved in the study.

## Role of funding source

The Foundation University Medicine Essen had no influence on the conducted study, the preparation of the manuscript, or the decision to submit this article for publication.

## Declaration of generative AI and AI-assisted technologies in the writing process

During the preparation of this work, the authors used ChatGPT (OpenAI) in order to improve linguistic quality. After using this tool, the authors reviewed and edited the content as needed and they take full responsibility for the content of the publication.

## CRediT authorship contribution statement

**Gwenllian A. Appeltrath:** Investigation, Methodology, Validation, Data curation, Formal analysis, Visualization, Writing – original draft, Writing – review & editing. **Janine Parreuter:** Resources, Writing – review & editing. **Monika Lindemann:** Resources, Investigation, Formal analysis, Writing – review & editing. **Hannes Klump:** Resources, Writing – review & editing. **Christina B. Karsten:** Conceptualization, Methodology, Validation, Formal analysis, Visualization, Supervision, Resources, Writing – original draft, Writing – review & editing.

## Declaration of competing interest

The authors declare that they have no known competing financial interests or personal relationships that could have appeared to influence the work reported in this paper.

## Data Availability

Data will be made available on request.
